# The University of Strasburg

**Published:** 1891-07-04

**Authors:** 


### The University of Strasburg.

An Alsatian will sometimes ask why so many Englishmen and Americans come to study in Strasburg. The fact is undeniable, for there were in the winter of 1889-90 thirteen Englishmen and forty Americans out of a total of some 900 students. They study the most diverse subjects; some organic chemistry, under Professor Fittig; some pharmacology and pharmacognosy, under Schmiedeberg and Flückiger; others Anglo-Saxon, under ten Brink; and yet others pathology, under Recklinghausen. Upon the whole, the reason is that Strasburg is, in many respects, a typical University town, and the professors men of reputation in English-speaking lands.

The city is not so large as to distract a student with head indifferently set upon his shoulders, or so small as to become monotonous. Half-an-hour with the tramway brings one to Kehl and the Rhine, neither picturesque nor suitable for rowing. There are other rivers near, dividing Strasburg so as to remind the traveller of Venice, and with stretches of water that an Oxford or Cambridge oarsman would view with envy. A boathouse has been built, with the adopted English word "club" written upon it, betokening that it is the premises of a rowing club, but the affair is of no importance. Bicycling is more indulged in, and bicyclists are occasionally met with on the road. Football-fields and cricket-grounds are not laid out, however suitable for these games the wide, level country might be, for the amusements of the German lie in quite a different direction. In the town there is a municipal theatre, a theatre or two of varieties, and innumerable cafés, restaurants, and bierhalles.

Since the war of '70, or simply '70, as the people call it, which leaves its traces still, not only in the little cemetery along the road to the Rhine, where relatives go in the autumn to deck the graves and burn candles when the Festival of the Dead comes round, but also in the city, a new University has taken the place of the former disjointed fragments. Remains of the French *régime* are still visible in the Public Library, with commemoration of Harvey, Jenner, and Sydenham, a tribute to Englishmen less usual on purely German soil, and in the Bürgerspital, which often goes by its French designation of the Civil Hospital, and is French in its buildings, appointments, and partly in its language; but the old names of streets are changed, the old University, with parts in four or five quarters of the town, is superseded, and a former professor is now M. Pasteur, of Paris.

When peace was signed in 1871 a sum of £2,400,000 was paid out of the war indemnity into the treasury of Strasburg, and with a part of this money the handsome block containing the public rooms of the University, opened May 1st, 1872, was erected. This principal building is fronted with ornamental grounds, and looks across a branch of the River Ill to the Kaiser's palace. Ten minutes away are the Zoological Schools, opposite the military exercise place, a part of the old French University, which had five faculties, dating from 1803. Quite in another quarter of the city, in close proximity to the Hospital, are a series of new blocks containing the physiological, anatomical, and other schools, together with the surgical clinic, the asylum, and the wards for diseases of women.

Turning round the Germania Restaurant, where many of the students dine daily at one o'clock, the central University stands full in view. On one side above the entrance is a statue of Argentina, in commemoration of the fact that Strasburg was once a Roman colony; on the other, a figure of Germania, with all round a series of statuettes of German savants and men of letters known only to the learned among ourselves, if we except the great traveller, Humboldt. Posting a letter in the letter-box hanging on the walls, as letter-boxes are placed in German towns, and entering, there are notices of medical, theological, and legal courses for the coming semester, or session, on the boards. This is one: "The laboratory will open on Monday, Oct. 28," signed Fittig; this another: "I shall begin a course of practical bacteriology early in January," signed Thierfelder, the assistant to Professor Hoppe-Seyler. There is a room with "Porter" over the doorway, where one may consult that functionary, partake of a small refreshment, or purchase at a cost of one halfpenny a dozen, or rather a "ten," for things run in tens in Strasburg, order forms for books from the University Library. In the left-letter rack are documents for "Herr Student of Philosophy This," or "Herr Student of Medicine That," with all their titles given in full. Letters for ladies there are none, for no ladies study in Strasburg, though there are a few in the freer air of Zurich, among them a daughter of the great German Socialistic leader, Herr Bebel. It was stated lately with authority that these ladies are very well received both in University and social circles in Switzerland, only that some Russians, with their careless attire and manners, brought discredit upon the whole body.

One comes upon a curious structure on mounting a few steps, in the form of a square area, paved in white and black irregular mosaic, running up to the height of both storeys of the University building, having handsome black marble pillars ranged at intervals round its four sides and roofed in with glass, partially in colours, which lies open to the sky. Across it, opposite to us, is the University reading-room, open to all matriculated students on payment of a terminal charge of four shillings. It contains daily and weekly, general and scientific journals from all quarters of the civilised world; and here the readers congregate when the day's work is over, about six o'clock in the evening. The purely medical journals have been removed to a separate reading-room, located in the Anatomical Schools. Last, but not least, there is in the reading-room the sale of theatre tickets, where students pay for an orchestral stall 1s. 6d., instead of the 3s. charged to the public. This is one of the privileges of the German University man.

